# Association of breast and gut microbiota dysbiosis and the risk of breast cancer: a case-control clinical study

**DOI:** 10.1186/s12885-019-5660-y

**Published:** 2019-05-24

**Authors:** Julio Plaza-Díaz, Ana I. Álvarez-Mercado, Carmen M Ruiz-Marín, Iris Reina-Pérez, Alejandro J. Pérez-Alonso, María Belén Sánchez-Andujar, Pablo Torné, Tania Gallart-Aragón, María Teresa Sánchez-Barrón, Saturnino Reyes Lartategui, Federico García, Natalia Chueca, Ana Moreno-Delgado, Katia Torres-Martínez, María José Sáez-Lara, Cándido Robles-Sánchez, Mariana F. Fernández, Luis Fontana

**Affiliations:** 1 Department of Biochemistry and Molecular Biology II, School of Pharmacy, University of Granada, Campus de Cartuja s/n, 18071 Granada, Spain; 2 Institute of Nutrition and Food Technology “José Mataix”, Biomedical Research Center, Parque Tecnológico Ciencias de la Salud, University of Granada, Granada, Spain; 3 Instituto de Investigación Biosanitaria ibs.GRANADA, Granada, Spain; 4 Spanish Consortium for Research on Physiopathology of Obesity and Nutrition (CIBEROBN), Instituto de Salud Carlos III, Madrid, Spain; 5 Unit of Mammary Pathology, General Surgery Service, University Hospital of Jaén, Jaén, Spain; 6 Department of Radiology, School of Medicine, University of Granada, Granada, Spain; 7 Spanish Consortium for Research on Epidemiology and Public Health (CIBERESP), Instituto de Salud Carlos III, Madrid, Spain; 8 Department of Surgery, School of Medicine, University of Granada, Granada, Spain; 9 Unit of Mammary Pathology, General Surgery Service, University Hospital Campus de la Salud, Granada, Spain; 10 Department of Microbiology, University Hospital Campus de la Salud, Granada, Spain; 11 Clínica Ana Moreno, Calle Doctor Alejandro Otero, Granada, Spain; 12 Department of Biochemistry and Molecular Biology I, School of Sciences, University of Granada, Granada, Spain; 13 Laboratory 207, Biomedical Research Center, Parque Tecnológico Ciencias de la Salud, Avda. del Conocimiento s/n, Armilla, 18100 Granada, Spain

**Keywords:** Archaea, Bacteria, Breast cancer, Breast microbiota, Endocrine disruptors, Environmental pollutants, Fungi, Gut microbiota, Virus

## Abstract

**Background:**

Breast cancer ranks first in women, and is the second cause of death in this gender. In addition to genetics, the environment contributes to the development of the disease, although the factors involved are not well known. Among the latter is the influence of microorganisms and, therefore, attention is recently being paid to the mammary microbiota. We hypothesize that the risk of breast cancer could be associated with the composition and functionality of the mammary/gut microbiota, and that exposure to environmental contaminants (endocrine disruptors, EDCs) might contribute to alter these microbiota.

**Methods:**

We describe a case-control clinical study that will be performed in women between 25 and 70 years of age. Cases will be women diagnosed and surgically intervened of breast cancer (stages I and II). Women with antecedents of cancer or advanced tumor stage (metastasis), or who have received antibiotic treatment within a period of 3 months prior to recruitment, or any neoadjuvant therapy, will be excluded. Controls will be women surgically intervened of breast augmentation or reduction. Women with oncological, gynecological or endocrine history, and those who have received antibiotic treatment within a period of 3 months prior to recruitment will also be excluded. Blood, urine, breast tissue and stool samples will be collected. Data regarding anthropometric, sociodemographic, reproductive history, tumor features and dietary habits will be gathered.

Metabolomic studies will be carried out in stool and breast tissue samples. Metagenomic studies will also be performed in stool and breast tissue samples to ascertain the viral, fungal, bacterial and archaea populations of the microbiota. Quantitation of estrogens, estrogen metabolites and EDCs in samples of serum, urine and breast tissue will also be performed.

**Discussion:**

This is the first time that the contribution of bacteria, archaea, viruses and fungi together with their alteration by environmental contaminants to the risk of breast cancer will be evaluated in the same study. Results obtained could contribute to elucidate risk factors, improve the prognosis, as well as to propose novel intervention studies in this disease.

**Trial registration:**

ClinicalTrials.gov NCT03885648, 03/25/2019. Retrospectively registered.

## Trial status

The trial is currently in progress. Enrolment of patients and analysis of data continues.

## Background

Humans are colonized by commensal microorganisms, which form the human microbiota. The community of microorganisms that exist within the gastrointestinal (GI) ecosystem is termed gut microbiota [1]. The GI microbiota is mainly composed by up to 10^14^ bacteria and several archaea, eukaryotes and virus [2]. In fact, humans are thought be in involved in a symbiotic relationship with the gut microbiota [3]. In human health, the function of the GI microbiota is to keep a dynamic equilibrium with the host, playing both local and remote roles [4] in important physiological processes, particularly inflammation and the immune response [2]. This interspecies balance is named eubiosis [5]. However, a “disequilibrium” in the microbiota (referred to as dysbiosis) may lead to several human diseases [4].

Given that the gastrointestinal tract is a complex, open, and integrated ecosystem with the external environment [5], exposure to external insults (i.e. pesticides and many other pollutants) may lead to dysbiosis. Failures of host barriers, defects in the immune system, and loss of eubiosis have been proposed to explain the potential impact of gut microbiota on carcinogenesis [6]. Moreover, it has been suggested that changes in the microbiota may even promote the development of extra-intestinal tumors [4] and be involved in its aggressiveness [7]. Recent findings revealing gut microbiota variations in patients with benign compared with malignant tumors suggest that the microbiome profile could be associated with cancerous state of the tumor [7].

Breast cancer ranks first and is the second cause of death in women [8]. In addition to genetics, environmental factors such as the influence of microorganisms could have a role in the development and progression of this disease [9, 10].

Although human breast tissue was previously presumed to be sterile, high-throughput technologies revealed a microbiome profile that has recently been characterized [7]. Furthermore, the local breast microbiota seems to differ in women with and without breast cancer [10].

Concerning the influence of microbes on breast cancer, some studies have focused on viruses. Up to 50% of breast tumors are Epstein-Barr positive [11], and human papilloma virus also appears to be associated with breast cancer [12]. In addition, the diversity and composition of the gut microbiota may affect breast cancer risk through the modulation of systemic levels of estrogens and inflammation [13]. In this sense, alterations of microbes able to metabolize estrogens and other endogenous hormones lead to an increment in circulating estrogen levels, which ultimately increase the risk of breast cancer development [14]. Furthermore, a reduced abundance of *Methylobacterium* in breast cancer patients has been correlated with tumors of greater invasive potential [15]. Moreover, exposure to a wide variety of xenobiotics (i.e. endocrine disruptors compounds (EDCs), which are found in routinely used products, lead to an increment of the risk of breast cancer [9]. Indeed, it has been hypothesized that in utero exposure to EDCs plays a role in breast cancer in women later in life [16]. Altogether, this background has drawn the attention of the scientific community in the mammary microbiome direction, albeit studies are still very limited and some of the published results are contradictory [17].

The rationale of our work is that the risk of breast cancer could be associated with the composition and functionality of the mammary/gut microbiota, and that exposure to environmental contaminants (i.e. EDCs) might contribute to alter both the gastrointestinal and mammary microbiota. Consequently, we aim to decipher the correlation among i) the composition of both the mammary and the gut microbiota, ii) the metabolic pathways in which they are involved, iii) the role of EDCs exposure to its alteration, and iv) the risk and progression of breast cancer, which might have broad implications in the prevention, diagnosis, treatment and prognosis of this disease.

## Methods/design

The study will follow the Declaration of Helsinki (52nd General Assembly, Edinburgh, Scotland, October 2000) and the Spaniard legislation regarding clinical research: Normas de Buena Práctica Clínica y Ley de Investigación Biomédica Real Decreto 561/1993 y 033/2004. All data obtained will be confidential and only the researchers and participants, upon request, will have access to them. The study will also follow the data protection legislation of Spain (Ley Orgánica de Protección de Datos de Carácter Permanente, Ley 15/1999 de 13 de Diciembre de 1999) to guarantee data confidentiality, treatment and availability for the participants.

Participation in the study is volunteer. Participants will be informed about the nature of the research and the usage of biological samples as well as the obtained data. In addition to verbal information, participants will be presented and read an informed consent. Permission has been granted by the Ethics Committee of Andalusia (Comité de Ética de la Investigación Biomédica de Andalucía, Spain).

### Study subjects

#### Inclusion and exclusion criteria

In this ongoing case-control study, already registered in ClinicalTrials.gov (Identifier NCT03885648), women’s age will range 25–70 years. Cases will be defined as women diagnosed and surgically intervened of incident breast cancer, stages I and II. Controls will be women surgically intervened of breast augmentation or reduction. Women with antecedents of cancer or advanced tumor stage (metastasis), or who have received antibiotic treatment within a period of 3 months prior to recruitment, or any neoadjuvant therapy, will be excluded of the study. Women with oncological, gynecological or endocrine antecedents and those who have received antibiotic treatment within a period of 3 months prior to recruitment will be also excluded. Controls will be matched to cases by age (± 2 years), category of body mass index and hospital of recruitment.

#### Sample size

Sample size has been calculated taking into consideration the incidence of breast cancer in Spain, the female population of Andalusia and the formula described by Aguilar-Barojas (2005) [18]. Because of that, the number of breast cancer cases will include at least 100 women, matched with 100 control women subjected to breast reduction or augmentation. Three hospital centers will participate in the recruitment: The Unit of Mammary Pathology, General Surgery Service, University Hospital of Jaén, Spain; the Unit of Mammary Pathology, General Surgery Service, University Hospital Campus de la Salud, Granada, Spain; and Ana Moreno Clinic (Granada, Spain).

#### Biological samples

Blood, breast tissue, stool and urine samples from cases and controls will be collected. Cases women will be enrolled on the same day of the diagnosis. They will be informed about the goals and procedures of the study and their participation will be requested, and those who accept to participate in the study will sign the informed consent. They will be supplied with a kit containing: 1 thermal bag, 1 freezer block, 1 pair of latex gloves, 1 disposable bedpan, 1 spatula, and 2 disposable plastic containers for urine and stool samples.

The day of the surgery, a blood sample will be taken. Serum will be separated by centrifugation to carry out a thorough biochemical analysis and determine estrogens. Breast tissue samples will be taken during surgery, from a marginal area 3–5 cm apart from the tumor. For controls, samples will be taken from any available breast tissue during breast reduction or augmentation surgery. Both feces and urine samples, from healthy and breast cancer-affected women will be collected the day previous to mammary surgery.

### Breast and intestinal microbiota

Bacteria, archaea, fungi and viruses will be investigated in feces and breast tissue samples. Samples will be pre-treated with pathogen lysis tubes L and S (QIAGEN, Barcelona, Spain). DNA will be extracted with the QIAamp cador Pathogen Mini kit (QIAGEN, Barcelona, Spain), following the manufacturer’s directions.

#### DNA quantification

The DNA concentration of the samples will be evaluated in a NanoDrop2000c (Thermo Fisher Scientific, Waltham, MA, USA).

#### Metagenomic library construction and sequencing

The Nextera XT DNA Library Preparation Kit (Illumina, San Diego, CA, USA) will be used for metagenomic library construction. The amplicon tagment mix (ATM) in Nextera XT, which includes the enzyme used for tagmentation, will be diluted 1:10 in nuclease-free water for library construction using 1 to 100 pg input DNA. Each 20 μL of the tagmentation reaction mixture consists of 10 μL TD buffer, 5 μL of input DNA, and 5 μL of diluted ATM. PCR cycles for library construction will be 12, 14, 17, and 20 cycles for 1000, 100, 10, and 1 pg DNA, respectively, following the manufacturer’s protocol. The manufacturer recommends 12 cycles of the PCR reaction for no less than 1 ng input DNA. Amplified libraries will be purified using AMPure XP (Agencourt, Brea, CA, USA). The quality of the purified libraries will be assessed using an Agilent High Sensitivity DNA Kit on an Agilent 2100 Bioanalyzer (Santa Clara, CA, USA). The sequencing libraries will be further quantified using the KAPA Library Quantification Kit. Metagenomic libraries will be mixed with PhiX Control v3 (Illumina) at a ratio of 9:1 and sequenced with an Illumina MiSeq Reagent Kit v3 (600 cycles).

All samples to be analyzed will be combined in a pool before starting the massive sequencing. The latter will be done with the MiSeq apparatus (Illumina).

#### Data processing for metagenomic libraries

Metagenomic reads will be subjected to adaptor clipping and quality trimming using Trimmomatic v0.33 [19]. The first three nucleotides with quality scores less than 20 will be cut from the 3′ and 5′ read ends. Reads will be processed using a sliding window method, cutting once the average quality within the window (4 base) fell below the threshold (Q20). Reads with a length of fewer than 100 nucleotides will be then removed. Low-complexity reads will be filtered out using PRINSEQ version 0.20.4 [20]. PCR duplicates will be removed with Picard version 2.8.0 (http://broadinstitute.github.io/picard). The processed high-quality and clean reads in each library will be used in subsequent analyses. In the community analysis based on metagenomic sequences, small subunit (SSU) rRNA gene sequences will be identified using Metaxa2 software [21]. Taxonomy assignments will be performed based on the results of a BLAST search against the SILVA123 database [22], using the MEGAN program [23] with the following settings: Min Support 1, Min Score 50, Max Expected 1xe^− 5^, Top Percent 10.0. SSU rRNA gene community compositions were compared among the metagenomic libraries.

### Functionality of the intestinal and mammary microbiota

Microbiota functionality will be investigated with a metabolomic study and by evaluating their capacity to damage the DNA of HeLa cells.

#### Metabolomic study

All the metabolomic analyses and data processing will be carried out with methods previously described [24–26].

##### Sample preparation

The samples will be prepared using the automated MicroLab STARR (Hamilton Company, Salt Lake City, UT, USA) robot system. For quality control (QC) purposes, a recovery standard will be added to 100 L of the serum samples before the first step in the extraction process. The proteins will be precipitated with methanol by vigorously shaking for 2 min. Next, the samples will be centrifuged to remove the proteins, dissociate the small molecules bound to the proteins or trapped in the precipitated protein matrix, and to recover chemically diverse metabolites (Glen Mills GenoGrinder 2000, Lebanon, USA). Then, the samples will be placed on a TurboVapR (Zymark, California, USA) to remove the organic solvent. For liquid chromatography (LC) analysis, the samples will be stored overnight in nitrogen before preparation. For gas chromatography (GC) analysis, each sample will be dried overnight under vacuum before preparation [24].

##### Quality controls

Several types of control experiments will be performed in parallel with the experimental samples. A pooled matrix sample, generated by taking a small volume of each experimental sample, will serve as a technical replicate throughout the extracted data set. Water aliquots will serve as process blanks. Moreover, a cocktail of QC standards carefully chosen not to interfere with the measurement of endogenous compounds will be spiked into every analyzed sample. This will allow instrument performance monitoring and aid in the chromatographic alignment. The instrument variability will be determined by calculating the median relative standard deviation (RSD) for the standards that will be added to each sample before injection into the mass spectrometers (MS). The entire process variability will be determined by calculating the median RSD for all the endogenous metabolites (i.e., non-instrument standards) present in 100% of the pooled matrix samples. The experimental samples will be randomized across the platform run with the QC samples spaced every 5 or 10 injections to verify overall assay performance [24]. The internal standards will be used to measure the instrument variability and will have a median RSD of 3%. Additionally, the total process variability will be 8%.

##### Ultra-performance liquid chromatography-tandem mass spectrometry

The liquid chromatography/mass spectrometry (LC/MS) portion of the platform to be used will employ a Waters ACQUITY ultra performance liquid chromatography (UPLC), a Thermo Scientific Q-Exactive high-resolution/accurate MS interfaced with a heated electrospray ionization (HESI-II) source, and an Orbitrap mass analyzer operated at a 35,000 mass resolution. The sample extract will be dried and reconstituted in acidic or basic LC-compatible solvents, each of which will contain eight or more injected QC standards at fixed concentrations to ensure injection and chromatographic consistency. One aliquot will be analyzed using acidic, positive ion-optimized conditions, and another will be performed using basic, negative ion optimized conditions in two independent injections using separate dedicated columns (Waters UPLC BEH C18–2.1 × 100 mm, 1.7 m). The extracts reconstituted in acidic conditions will be gradient-eluted from a C18 column using water and methanol containing 0.1% formic acid. A second aliquot, from the basic extract, will be similarly eluted from a C18 column using methanol, water, and 6.5 mM ammonium bicarbonate. A third aliquot will be analyzed via negative ionization following elution from a hydrophilic interaction chromatography column (Waters UPLC BEH Amide 2.1 × 150 mm, 1.7 m) using a gradient consisting of water and acetonitrile with 10 mM ammonium formate [24].

##### Gas chromatography-mass spectrometry

The samples destined for analysis by GC-MS will be dried under a vacuum for a minimum of 18 h before being derivatized under dried nitrogen with bistrimethylsilyltrifluoroacetamide. The derivatized samples will be separated on a 5% diphenyl/95% dimethyl polysiloxane fused silica column (20 m × 0.18 mm id; 0.18 m film thickness) with helium as the carrier gas and a temperature ramp from 60C to 340C over a 17.5-min period. The samples will be analyzed on a Thermo-Finnigan Trace DSQ fast-scanning single-quadrupole MS using electron impact ionization (EI) operated at unit mass resolving power. The scan range will be 50–750 m/z [24].

##### Data extraction and compound identification

The raw data will be extracted, and the peaks will be aligned and identified. QCs will be processed using specific hardware and software [25]. The peaks will be quantified using the area-under-the-curve (AUC) calculation. The compounds will be identified comparing the data to library entries of purified standards or recurrent unknown entities. The biochemical identifications will be based on three criteria: the retention index (RI) within a narrow RI window of the proposed identification, an accurate mass match to the library ±0.005 amu, and the MS/MS forward and reverse scores between the experimental data and authentic standards. The MS/MS scores will be based on a comparison of the ions present in the experimental spectrum with the ions present in the library spectrum. Although there may be similarities between these molecules based on one of these factors, the use of all three data points can be utilized to distinguish and differentiate the biochemicals with precision.

#### Evaluation of DNA damage by determination of phosphorylated histone 2AX (P-H2AX)

Breast biopsies will be homogenized and seeded onto specific culture media to isolate bacterial colonies which later on will be evaluated based on their ability to generate nicks in the DNA of HeLa cells using the P-H2AX marker [27]. For this purpose, after colony isolation an under both aerobic and anaerobic conditions, bacterial species will be identified by PCR amplification of 16S rRNA gene. Then bacterial candidates will be used to infect HeLa cells in 24 well-dishes for 24 h. Cells will be fixed and subjected to immunofluorescence using an anti-phosphorylated histone 2AX (P-H2AX, rabbit anti-phosphohistone-2AX MAb; Cell Signaling Technologies, ref. 9718). Fluorescence intensity will be measured and the percentage of P-H2AX positive cells will be calculated.

### Quantitation of the exposure to EDCs in urine samples

Analysis of non-persistent EDCs will be carried out by dispersive liquid–liquid microextraction (DLLME) and ultra-high performance liquid chromatography with tandem mass spectrometry detection (UHPLC-MS/MS) as previously described with minor modifications [28, 29]. Briefly, urine samples will be thawed completely at room temperature, centrifuge at 2600 x g for 10 min to sediment particulate matter and 0.75 mL will be taken to carry out the analysis. In order to determine total EDCs amount (free plus conjugated) in urine, each sample will be spiked with 50 μL of enzyme solution (β-glucuronidase/sulfatase) and incubated at 37 °C for 24 h. The treated urine will be placed in a 15 mL screw-cap glass tube and spiked with 30 μL of the surrogate standard solution (1.25 mg/L of BPA-d_16_). Urine will be diluted to 10.0 mL with 5% NaCl aqueous solution (w/v) and the pH will be adjusted to 2.0. Next, 0.75 mL of acetone and 0.75 mL of trichloromethane will be mixed and injected rapidly into the aqueous sample with a syringe. After manual shaking, centrifugation and evaporation of the extract, the residue will be dissolved with 100 μL of a mixture consisting of water (0.1% ammonia)/acetonitrile (0.1% ammonia), 70:30 (v/v), and finally 10 μL will be injected in the LC system. Urinary creatinine concentration (mg/dL) will be determined using an automated colorimetric determination, in the same urine samples in which environmental chemical will be assessed. Because of the relatively constant excretion rate of creatinine into the urine (which makes urinary creatinine concentration inversely proportional to urine flow rate), creatinine adjustment is widely used to normalize analyte concentrations in spot samples for environmental exposure monitoring [30].

Bisphenols [bisphenol A (BPA), bisphenol S (BPS) and bisphenol F (BPF)], parabens [(metylparaben (MeP), ethylparaben (EtP) propylparaben (PrP) and butylparaben (BuP)], benzophenones [(UV filters: benzophenone-1 (BP-1), benzophenone-3 (BP-3) and 4-hidroxybenzophenone (4-OH-BP)] will be determined. Final concentrations will be adjusted for urinary creatinine levels (mg/dl), to account for both urine dilution and body composition, as previously reported [31, 32].

### Quantitation of urinary concentrations of the parent estrogens and estrogen metabolites

Concentrations of estrone, estradiol and 13 estrogen metabolites [2-hydroxylated estrogen metabolites (2-hydroxyestrone, 2-methoxyestrone, 2-hydroxyestradiol, 2-methoxyestradiol, and 2-hydroxyestrone-3-methyl ether); 4-hydroxylated estrogen metabolites (4-hydroxyestrone, 4-methoxyestrone, and 4-methoxyestradiol); and 16-hydroxylated estrogen metabolites (16α-hydroxyestrone, estriol, 17-epiestriol, 16-ketoestradiol, and 16-epiestriol)] will be determined in fasting spot urine samples, adjusting for urinary creatinine levels (mg/dL). The quantitation of urinary estrogens will be assessed by gas chromatography tandem mass spectrometry using a triple quadrupole analyzer as previously described with minor modifications [33, 34]. Briefly, one mL of urine with 25 μL of internal standards solution will be extracted using C18 SPE cartridge (Sep-Pack C18) previously conditioned with 4 mL of methanol and 4 mL of water. After loading the urine sample, the cartridge will be washed with 4 mL of water. The treated urine will then be evaporated. Three mL of acetate buffer 0.1 M (pH 4.5) and 3 μL of β-glucuronidase will be added to the treated urine after being evaporated, and the mixture will be incubated for 3 h at 55 °C. After cooling to room temperature, pH will be increased to approximately 9.5. Then, samples will be extracted with 6 mL of tert-butyl methyl ether by shaking in a rocking mixer and centrifuged. Finally, the organic layer will be evaporated to dryness under a stream of nitrogen. Two μL of the derivatized extract will be injected in split mode into the GC-MS/MS system. All hormone assessments will be carried out simultaneously to reduce intralaboratory variation.

### Other sources of information and covariables

Participants’ anthropometric, sociodemographic and reproductive features will be recorded. They will also grant permission to access their clinical history.

#### Anthropometric and sociodemographic information

Age, race, weight, height, residence area (rural/urban), marital status, education, current working activity, social class, smoking habits (never, current, former), and alcohol consumption (never, current, past), will be recorded.

#### Reproductive history

Age of menarche and menopause status (premenopause, postmenopause), number of pregnancies, number of sons, age at the first and last pregnancy, total duration of breast feeding, use of hormonal contraceptives and/or substitute hormonal therapy (age at beginning and duration).

#### Tumor features

Type and hystological grade according to the International Classification of Oncological Diseases (CIE-0-2); tumor stage: clinical and anatomopathogical TNM (malignant tumor classification); hormone receptors (estrogens and progesterone); HER2, and tumor markers (pS2, cathepsin D and p53).

#### Diet

Participants will answer two questionnaires: adherence to the Mediterranean diet and frequency of food consumption.

### Statistical analysis

Relative abundance of the microbes will be expressed as median ± range. Differences among microbes will be calculated by the *U*-Mann de Whitney. Intestinal and mammary microbiota clusters will be calculated by an analysis of principal components made with the bioinformatic data. Those data will be normalized between 0 and 1, and the Euclidean distance will be evaluated. A permutation ANOVA (PERMANOVA) will be carried out with the PRIMER 7 software. Finally, a tree diagram will be built for each evaluated group based on the different taxonomic scales.

For metabolomic statistical analysis and data display purposes, any missing values will be assumed to be below the limits of detection, and these values will be imputed with the compound minimum (minimum value imputation). The data will be tested for variance homogeneity, and if there were unequal variances, the data will be log-transformed. The statistical analysis of the data will be performed using Array Studio (OmicSoft, NC, USA) and proprietary software from Metabolon. A t-test for equality of the means will be carried out between the intestinal and mammary microbiota and for each metabolite to determine differences. A *p*-value 0.05 will be considered statistically significant. A one-way ANOVA, assuming homogeneity of variance, will be used to determine whether at least two unknown means are all equal or at least one pair of means is different. In case that the variances were unequal, a Welch-ANOVA will be performed. The false discovery rate (q-value) will be calculated to take into account the multiple comparisons that normally occur in metabolomics-based studies, thus estimating the reliability of the results. A q-value 0.1 will be considered statistically significant.

Random forest (RF) is a supervised classification technique based on an ensemble of decision trees [35] with several advantages. It makes no parametric assumptions, and variable selection is not needed. It does not overfit, and it is invariant to transformation. In RF analysis, it is not necessary to cross-validate or do a separate test set to obtain an unbiased estimate of the test set error. A “variable importance” measure will be computed to determine which variables (metabolites) make the largest contribution to the classification. The mean decrease in accuracy (MDA) will be used as this metric and will be determined by randomly permuting a variable, running the observed values through the trees, and then reassessing the prediction accuracy. If a variable were not significant, then this procedure will have little change in the accuracy of the class prediction (permuting random noise will give random noise). By contrast, if a variable is relevant for the classification, the prediction accuracy will drop after such a permutation, which will be recorded as the MDA. Thus, the RF analysis provides an “importance” rank ordering for biochemicals [35].

Participants will be grouped into tertiles of urinary EDC levels based on their distributions among the controls. Odds ratios and 95% confidence intervals (CIs) for breast cancer comparing the second and third tertiles with the first tertile will be estimated using logistic regression models. Further, associations of participant characteristics with urinary EDC levels, estrogens and estrogen metabolites and fecal microbiome measures will be evaluated by using general linear models and testing either for differences across categories or for trends across categories. Linear regression models will also be used to evaluate associations of each steroid measure with measures of the diversity of the fecal microbiome, abundance and with taxonomic groups at phylum and family levels, adjusting by covariates.

## Discussion

There are 7 studies registered in ClinicalTrials.gov dealing with breast cancer and microbiota, 2 already completed and 4 currently ongoing. Of the 2 completed studies, one investigated the antibiotic profilaxis in oncological surgery of the breast, and the other investigated the relationship between intestinal bacteria and breast cancer risk. As for the studies in progress, these are trials investigating various intervention strategies (probiotics, physical activity, diet), the effects of gut microbiome on the neoadjuvant chemotherapy-induced immunosurveillance in triple negative breast cancer, or the effects of chemotherapy on gut bacteria in newly diagnosed breast cancer. Our study is a case-control clinical trial that aims at ascertaining the connection relating i) the composition of both gut and breast microbiota, ii) the functionality of both gut and breast microbiota, and iii) the exposure to environmental contaminants with the risk of developing breast cancer. To the best of our knowledge, it is the first study undertaking the simultaneous analysis of the microbiota of the mammary tissue and the intestine, and also the first attempt to link the microbiome with the EDC load and the risk of this disease.

The microbiota is affected by either modifiable and unmodifiable factors such as genetics, stress, diet, physical activity, lactation, delivery mode, antibiotic treatment, smoking, age, and alcohol consumption, among others [36]. For this reason, in an attempt to control some of the modifiable factors, all participants will answer three questionnaires to record their sociodemographic data, adherence to the Mediterranean diet, and frequency of food consumption. For the same reason, we set stringent inclusion and exclusion criteria. Women participating in the study will be newly diagnosed cases of breast cancer. Therefore, women with antecedents of cancer or advanced tumor stage will not take part, nor those who have received antibiotic treatment 3 months prior to recruitment, or any neoadjuvant therapy. Likewise, regarding the controls, women with oncological, gynecological or endocrine antecedents and those who have received antibiotic treatment 3 months prior to recruitment will not be included.

The hypothesis of this study is that the risk of breast cancer could be associated with the composition and functionality of the mammary and/or intestinal microbiota. The hypothesis is supported by preliminary studies which suggest that the microbial pattern of women with breast cancer is different than that of healthy women, not only with regards to bacterial types and relative abundance, but also with their functionality (metabolic capacity, ability y to damage DNA, etc.) [27, 37–39]. Exogenous factors such as the exposure to EDCs might contribute to this altered pattern [40, 41]. These circumstances would attribute to the microbiota the possibility of acting as an additional environmental risk factor and a potential prognostic modulator of the disease.

Validation of the hypothesis could translate into new recommendations and guidelines of clinical practice to improve prevention and prognosis of breast cancer, alleviating the burden of this disease. Likewise, a better identification of those patients most likely to efficiently respond to treatment could derive, given the fact that efficacy of immunotherapy against cancer depends on patients’ intestinal microbiota [42, 43]. Finally, interventional clinical studies aimed at shifting the mammary/gut dysbiosis of women affected by breast cancer toward a composition similar to that of healthy women could be designed.

## Abbreviations

4-OH-BP: 4-hidroxybenzophenone
AUC: Area-under-the-curve
BFS: Bisphenol S
BP-1: Benzophenone-1
BP3: Benzophenone-3
BPA: Bisphenol A
BPF: Bisphenol F
BuP: Butylparaben
CIE-0-2: Classification of Oncological Diseases
DLLME: Dispersive liquid–liquid microextraction
EDCs: Endocrine disruptors
EI: Electron impact ionization
EtP: Ethylparaben
GC: Gas chromatography
GI: Gastrointestinal
LC: Liquid chromatography
MDA: Mean decrease in accuracy
MeP: Metylparaben
MS: Mass spectrometers
PCR: Polymerase chain reaction
PERMANOVA: A permutation ANOVA
P-H2AX: Phosphorylated histone 2AX
PrP: Propylpaaben
QC: Quality control
RF: Random forest
RI: Retention index
RSD: Relative standard deviation
TNM: Tumor (T), nodes (N), and metastases (M)
UHPLC-MS/MS: Ultra-high performance liquid chromatography with tandem mass spectrometry detection
UPLC: Ultra performance liquid chromatography
UV: Ultraviolet
